# Comparative prognostic value of different preoperative complete blood count cell ratios in patients with oral cavity cancer treated with surgery and postoperative radiotherapy

**DOI:** 10.1002/cam4.3738

**Published:** 2021-02-23

**Authors:** Yao‐Yu Wu, Kai‐Ping Chang, Tsung‐Ying Ho, Wen‐Chi Chou, Sheng‐Ping Hung, Kang‐Hsing Fan, Yin‐Yin Chiang, Yung‐Chih Chou, Ngan‐Ming Tsang

**Affiliations:** ^1^ Department of Radiation Oncology Linkou Chang Gung Memorial Hospital and Chang Gung University Taoyuan City Taiwan; ^2^ Department of Otorhinolaryngology, Head and Neck Surgery Linkou Chang Gung Memorial Hospital and Chang Gung University Taoyuan City Taiwan; ^3^ Department of Nuclear Medicine and Molecular Imaging Center Linkou Chang Gung Memorial Hospital and Chang Gung University Taoyuan City Taiwan; ^4^ Division of Hematology‐Oncology Department of Internal Medicine Linkou Chang Gung Memorial Hospital and Chang Gung University Taoyuan City Taiwan; ^5^ School of Traditional Chinese Medicine Chang Gung University Taoyuan City Taiwan

**Keywords:** distant metastasis, lymphocyte‐to‐monocyte ratio, neutrophil‐to‐lymphocyte ratio, oral cavity squamous cell carcinoma, overall survival, platelet‐to‐lymphocyte ratio

## Abstract

**Background:**

We sought to compare the prognostic significance of different preoperative complete blood count cell ratios in patients with oral cavity squamous cell carcinoma (OSCC) treated with surgery and postoperative radiotherapy (PORT).

**Methods:**

We retrospectively reviewed the clinical records of 890 patients with OSCC who were treated with surgery and PORT. The following preoperative complete blood count cell ratios were collected: neutrophil‐to‐lymphocyte ratio (NLR), platelet‐to‐lymphocyte ratio (PLR), and lymphocyte‐to‐monocyte ratio (LMR). Overall survival (OS), local control, regional control, and distant control (DC) served as the main outcomes of interest.

**Results:**

The results of multivariate analysis in the entire study cohort revealed that a low NLR was the only independently favorable marker of both OS (adjusted hazard ratio [HR]: 0.794, 95% confidence interval (CI): 0.656–0.961, bootstrap *p* = 0.028) and DC (adjusted HR: 0.659, 95% CI: 0.478–0.909, bootstrap *p* = 0.015). Both LMR and PLR were not retained in the model as independent predictors. Subgroup analyses in high‐risk patients (i.e., those bearing T4 disease, N3 disease, or poor differentiation) revealed that a high NLR was a significant adverse risk factor for both OS and DC (all *p* < 0.03)—with a borderline significance being evident for DC in patients with T4 disease (*p* = 0.058).

**Conclusions:**

A high pretreatment NLR was an independent unfavorable risk factor for both OS and DC in patients with OSCC who underwent surgery and PORT. No other preoperative complete blood count parameters and cell ratios were found to have prognostic significance.

## Graphical abstract

This relatively large cohort reports a comparative result: pretreatment high neutrophil‐to‐lymphocyte ratio (NLR) was an independent unfavorable risk factor for both overall survival and distant metastasis in patients with oral cavity squamous cell carcinoma (OSCC) who underwent surgery and postoperative radiotherapy. Lymphocyte‐to‐monocyte ratio (LMR) and platelet‐to‐lymphocyte ratio (PLR) were not significant.

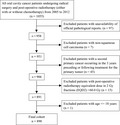

## 1 INTRODUCTION

Inflammation has been shown to promote tumor initiation and progression, whereas escape from immune surveillance may favor cancer invasiveness and distant spread.^1^, ^2^, ^3^ There is consistent evidence that a high tumor infiltration by neutrophils and macrophages has an adverse prognostic significance.^4^, ^5^ In contrast, tumor‐infiltrating lymphocytes portend more favorable outcomes.^6^, ^7^, ^8^ Various preoperative complete blood count cell ratios—including neutrophil‐to‐lymphocyte ratio (NLR), platelet‐to‐lymphocyte ratio (PLR), and lymphocyte‐to‐monocyte ratio (LMR)—have been extensively investigated in relation to prognosis in patients with different solid malignancies, including oral squamous cell carcinoma (OSCC).^9^, ^10^, ^11^, ^12^ However, the comparative value of NLR, PLR, and LMR for predicting clinical outcomes in patients with OSCC remains unclear. Further, most published studies have been focused on overall survival (OS).

Starting from these premises, we designed this study to specifically compare the prognostic significance of different preoperative complete blood count cell ratios in patients with OSCC treated with surgery and postoperative radiotherapy (PORT). Besides OS, the ratios were investigated in relation to other clinical endpoints—including local control (LC), regional control (RC), and distant control (DC).

## 2 MATERIALS AND METHODS

### 2.1 Patients

**FIGURE 1:**
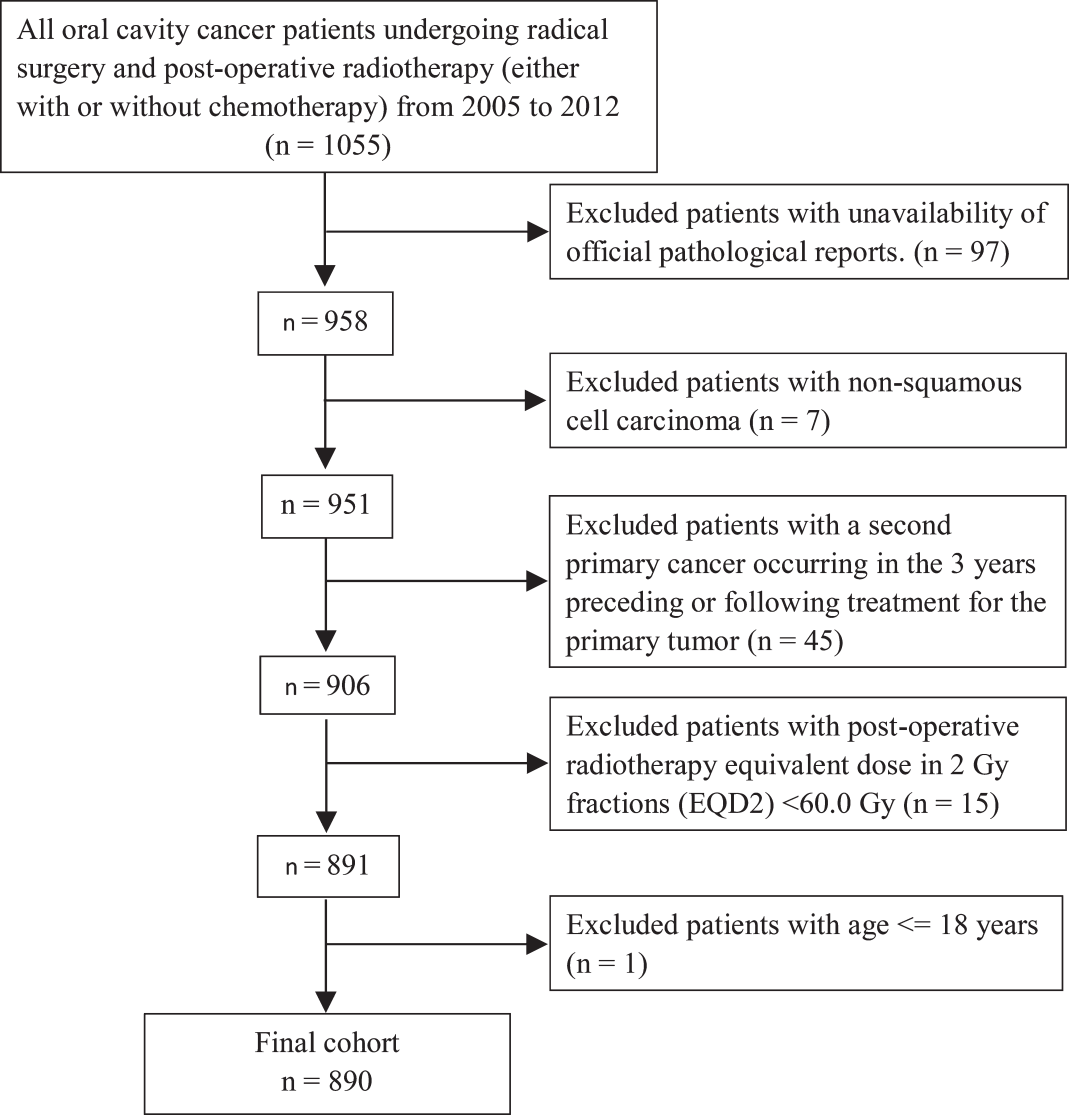
Patient flow chart of the study

We retrospectively reviewed the clinical records of patients who had undergone radical surgery and PORT (either with or without chemotherapy) at our hospital between January 2005 and December 2012 (*n* = 1055). Patient staging was performed according to the 2018 American Joint Committee on Cancer TNM staging system. Exclusion criteria were as follows: (a) unavailability of official pathological reports (*n* = 97), (b) not squamous cell carcinoma (*n* = 7), (c) presence of a second primary cancer occurring in the three years preceding or following treatment for the primary tumor (*n* = 45), (d) equivalent dose in 2 Gy fractions (EQD2) <60.0 Gy (*n* = 15), and 5) age <18 years (*n* = 1). Figure 1 depicts the flow of patients through the study. Data collection was performed by a radiation oncologist and an experienced nurse. The study protocol followed the tenets of the Helsinki declaration and was granted ethics approval by the Institutional Review Board Committee of our hospital. Owing to the retrospective nature of the study, the need for informed consent was waived.

### 2.2 Calculation of pretreatment blood count cell ratios

A pretreatment complete blood count was obtained in the 14 days preceding radical surgery. The three ratios of interest (NLR, PLR, and LMR) were calculated from absolute counts of neutrophils, lymphocytes, platelets, and monocytes, as appropriate.

### 2.3 Variable definitions

In keeping with the American Centers for Disease Control and Prevention classification system, cigarette smoking was dichotomized as yes (subjects who smoked ≥100 cigarettes in their lifetime) vs. no (subjects who smoked <100 cigarettes in their lifetime and not currently smoking). Alcohol drinking (current or former drinkers vs. non‐drinkers) and betel quid chewing (current or former chewers vs. nonchewers) were similarly considered as dichotomous variables. Pretreatment BMI—calculated as pretreatment weight in kilograms divided by height in meters squared—was dichotomized as underweight or normal (BMI <25 kg/m^2^) vs. overweight (BMI ≥25 kg/m^2^).

### 2.4 Statistical analyses

The primary outcome measure was OS, whereas LC, RC, and DC served as secondary endpoints. Survival was calculated as the time elapsed (in years) from the start of PORT to the event of interest. The optimal cutoff points for NLR, PLR, and LMR were based on where the Youden index (sensitivity +specificity – (a) was maximal via time‐dependent receiver operating characteristic (TDROC) curve analysis taking the overall survival (OS) at 5 years from the start of PORT as the endpoint of interest. Patients were divided into two groups (high vs. low) according to the optimal cutoff values. Intergroup differences were assessed with the Student's *t*‐test (continuous variables) or the chi‐square tests (categorical variables). Survival curves were constructed using the Kaplan‐Meier method (log‐rank test). Cox proportional hazard regression models were used to assess the impact of each variable on the study endpoints. Results are expressed as hazard ratios (HRs) with their 95% confidence intervals (CIs). Two‐tailed *p* values <0.05 were considered statistically significant. The bootstrap method (1000 resamples) was used for internal validation. TDROC curve analysis was performed in the R environment using the “timeROC” package (R Foundation for Statistical Computing, Vienna, Austria) through inverse probability of censoring weighting (IPCW) approach to estimate time‐dependent ROC curve and AUC for censored events with competing risks. All other calculations were carried out with SPSS, version 22.0 (IBM).

## 3 RESULTS

### 3.1 Optimal cutoff values for NLR, LMR, and PLR

TDROC curve analysis revealed that the areas under the curve for NLR, LMR, and PLR were 0.541, 0.540, and 0.542, respectively (Table S1). The optimal cutoff values for NLR, LMR, and PLR at time point of 5 years after surgery were as follows: 2.90, 4.21, and 110.6, respectively (Table S2). There were 567 (63.7%) patients with a low (<2.90) and 323 (36.3%) with a high NLR (≥2.90), respectively. With regard to LMR, 359 patients (40.3%) were in the low (<4.21) and 531 (59.7%) in the high (≥2.90) group, respectively. Finally, there were 354 (39.6%) patients with a low (<110.6) and 536 (60.2%) with a high PLR (≥110.6).

### 3.2 Patient characteristics

The general characteristics of the entire study cohort and different NLR, LMR, and PLR subgroups are summarized in Table 1. The median follow‐up time for patients who survived was 72.7 months (interquartile range: 14.9−101.4 months), whereas the median age of the study participants was 50.8 years (interquartile range: 44.2−57.8 years). Most patients (93.0%) were men. All participants received adjuvant radiation therapy and 533 (59.9%) were concurrently treated with chemotherapy. The most common primary tumor sites were the tongue (36.3%), buccal mucosa (35.4%), and gum (15.5%). NLR was associated with T stage (*p* < 0.001), N stage (*p* = 0.002), clinical stage (*p* < 0.001), and BMI (*p* = 0.002). Conversely, LMR was associated with sex (*p* = 0.007), T4 (*p* < 0.001), clinical stage (*p* = 0.003), and BMI (*p* < 0.001). A borderline association with smoking was also observed (*p* = 0.047). Finally, PLR was associated with T stage (*p* < 0.001), clinical stage (*p* = 0.01), and BMI (*p* < 0.001).

**TABLE 1:** General characteristics of the study participants

		NLR				LMR				PLR			
Variable	Entire cohort	Low (< 2.90)	High (≥ 2.90)			Low (< 4.21)	High (≥ 4.21)			Low (<110.6)	High (≥ 110.6)		
	Total (*n* = 890)	*n* = 567 (63.7)	*n* = 323 (36.3)		*p*	*n* = 359 (40.3)	N = 531 (59.7)		*p*	*n* = 354 (39.8)	*n* = 536 (60.2)		*p*
Age, years					0.407				0.400				0.313
< 60	721 (81.0)	464 (81.8)	257 (79.6)			286 (79.7)	435 (81.9)			281 (79.4)	440 (82.1)		
≥ 60	169 (19.0)	103 (18.2)	66 (20.4)			73 (20.3)	96 (18.1)			73 (21.6)	96 (17.9)		
Sex					0.020				0.007				0.210
Female	62 (7.0)	48 (8.5)	14 (4.3)			15 (4.2)	47 (8.9)			20 (5.6)	42 (7.8)		
Male	828 (93.0)	519 (91.5)	309 (95.7)			344 (95.8)	484 (91.1)			334 (94.4)	494 (92.2)		
Primary tumor site					0.380				0.454				0.222
Buccal mucosa	315 (35.4)	188 (33.2)	127 (39.3)			140 (39.0)	175 (33.0)			120 (33.9)	195 (36.4)		
Gum	138 (15.5)	87 (15.3)	51 (15.8)			59 (16.4)	79 (14.9)			55 (15.5)	83 (15.5)		
Hard palate	16 (1.8)	8 (1.4)	8 (2.5)			6 (1.7)	10 (1.9)			2 (0.6)	14 (2.6)		
Lip	18 (2.0)	13 (2.3)	5 (1.5)			6 (1.7)	12 (2.3)			7 (2.0)	11 (2.1)		
Mouth floor	33 (3.7)	23 (4.1)	10 (3.1)			14 (3.9)	19 (3.6)			17 (4.8)	16 (3.0)		
Retromolar	47 (5.3)	32 (5.6)	15 (4.6)			18 (5.0)	29 (5.5)			17 (4.8)	30 (5.6)		
Tongue	323 (36.3)	216 (38.1)	107 (33.1)			116 (32.3)	207 (39.0)			136 (38.4)	187 (34.9)		
AJCC 2018 T stage					<0.001				<0.001				<0.001
T_1_	30 (3.4)	26 (4.6)	4 (1.2)			4 (1.1)	26 (4.9)			11 (3.1)	19 (3.5)		
T_2_	74 (8.3)	59 (10.4)	15 (4.6)			21 (5.8)	53 (10.0)			37 (10.5)	37 (6.9)		
T_3_	409 (46.0)	287 (50.6)	122 (37.8)			141 (39.3)	268 (50.5)			186 (52.5)	223 (41.6)		
T_4_	377 (42.4)	195 (34.4)	182 (56.3)			193 (53.8)	184 (34.7)			120 (33.9)	257 (47.9)		
AJCC 2018 N stage					0.002				0.098				0.437
N_0_	325 (36.5)	192 (33.9)	133 (41.2)			141 (39.3)	184 (34.7)			130 (36.7)	195 (36.4)		
N_1_	148 (16.6)	112 (19.8)	36 (11.1)			48 (13.4)	100 (18.8)			67 (18.9)	81 (15.1)		
N_2_	180 (20.2)	122 (21.5)	58 (18.0)			68 (18.9)	112 (21.1)			69 (19.5)	111 (20.7)		
N_3_	237 (26.6)	141 (24.9)	96 (29.7)			102 (28.4)	135 (25.4)			88 (24.9)	149 (27.8)		
AJCC 2018 stage					<0.001				0.003				0.010
I	8 (0.9)	6 (1.1)	2 (0.6)			1 (0.3)	7 (1.3)			5 (1.4)	3 (0.6)		
II	19 (2.1)	17 (3.0)	2 (0.6)			5 (1.4)	14 (2.6)			9 (2.5)	10 (1.9)		
III	233 (26.2)	170 (30.0)	63 (19.5)			75 (20.9)	158 (29.8)			111 (31.4)	122 (22.8)		
IV	630 (70.8)	374 (66.0)	256 (79.3)			278 (77.4)	352 (66.3)			229 (64.7)	401 (74.8)		
Differentiation					0.629				0.526				0.200
Well	219 (24.6)	134 (23.6)	85 (26.3)			95 (26.5)	124 (23.4)			86 (24.3)	133 (24.8)		
Moderate	549 (61.7)	356 (62.8)	193 (59.8)			218 (60.7)	331 (62.3)			228 (64.4)	321 (59.9)		
Poor	122 (13.7)	77 (13.6)	45 (13.9)			46 (12.8)	76 (14.3)			40 (11.3)	82 (15.3)		
BMI, kg/m^2^					0.002				<0.001				<0.001
< 25	495 (55.6)	293 (51.7)	202 (62.5)			225 (62.7)	270 (50.8)			153 (43.2)	342 (63.8)		
≥ 25	395 (44.4)	274 (48.3)	121 (37.5)			134 (37.3)	261 (49.2)			201 (56.8)	194 (36.2)		
Cigarette smoking					0.396				0.047				0.523
No	116 (13.0)	78 (13.8)	38 (11.8)			37 (10.3)	79 (14.9)			43 (12.1)	73 (13.6)		
Yes	774 (87.0)	489 (86.2)	285 (88.2)			322 (89.7)	452 (85.1)			311 (87.9)	463 (86.4)		
Betel quid chewing					0.154				0.085				0.444
No	197 (22.1)	134 (23.6)	63 (19.5)			69 (19.2)	128 (24.1)			83 (23.4)	114 (21.3)		
Yes	693 (77.9)	433 (76.4)	260 (80.5)			290 (80.8)	403 (75.9)			271 (76.6)	422 (78.7)		
Alcohol drinking					0.338				0.542				0.618
No	318 (35.7)	196 (34.6)	122 (37.8)			124 (34.5)	194 (36.5)			123 (34.7)	195 (36.4)		
Yes	572 (64.3)	371 (65.4)	201 (62.2)			235 (65.5)	337 (63.5)			231 (65.3)	341 (63.6)		
Concurrent chemotherapy					0.838				0.675				0.069
No	357 (40.1)	226 (39.9)	131 (40.6)			141 (39.3)	216 (40.7)			155 (43.8)	202 (37.3)		
Yes	533 (59.9)	341 (60.1)	192 (59.4)			218 (60.7)	315 (59.3)			199 (56.2)	334 (62.3)		

Abbreviations: AJCC, American joint committee on cancer; BMI, body mass index; LMR, lymphocyte‐to‐monocyte ratio; NLR, neutrophil‐to‐lymphocyte ratio; PLR, platelet‐to‐lymphocyte ratio.

### 3.3 Preoperative complete blood count cell ratios and overall survival

**FIGURE 2:**
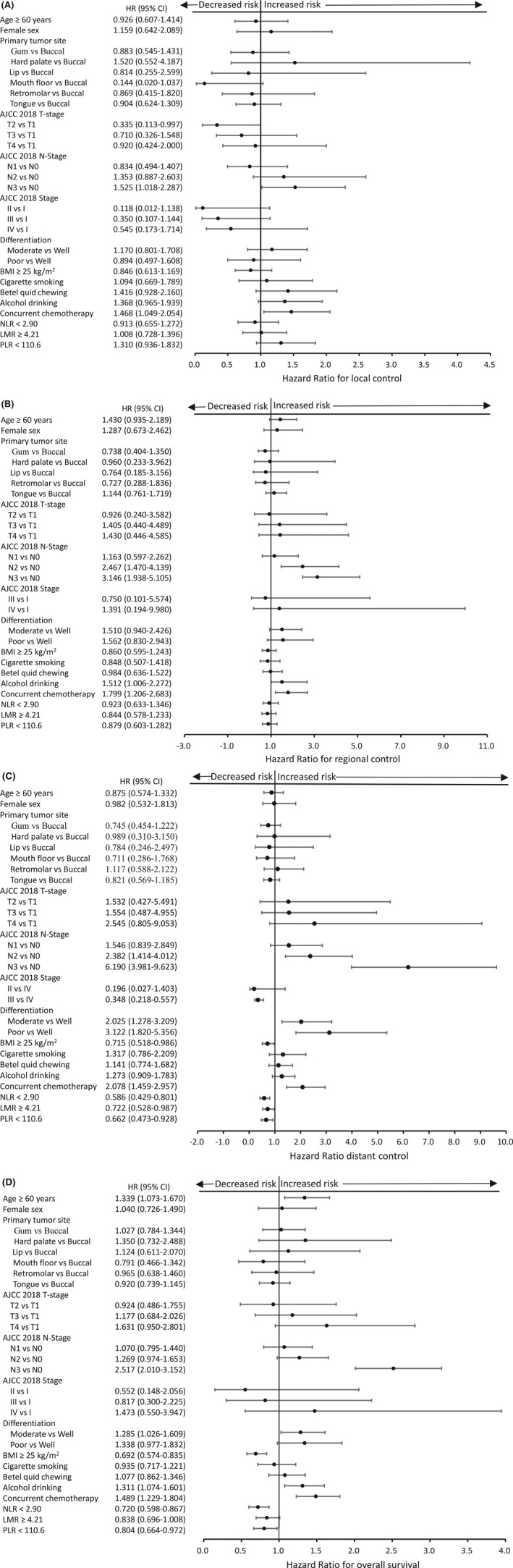
Forest plot based on univariate hazard ratios (HRs) from Cox regression for all variables. (A) local control (B) regional control (C) distant control and (D) overall survival in the entire cohort (*n* = 890). Abbreviations: AJCC, American Joint Committee on Cancer; BMI, body mass index; CI, confidence interval; HR, hazard ratio; LMR, lymphocyte‐to‐monocyte ratio; NLR, neutrophil‐to‐lymphocyte ratio; PLR, platelet‐to‐lymphocyte ratio

**FIGURE 3:**
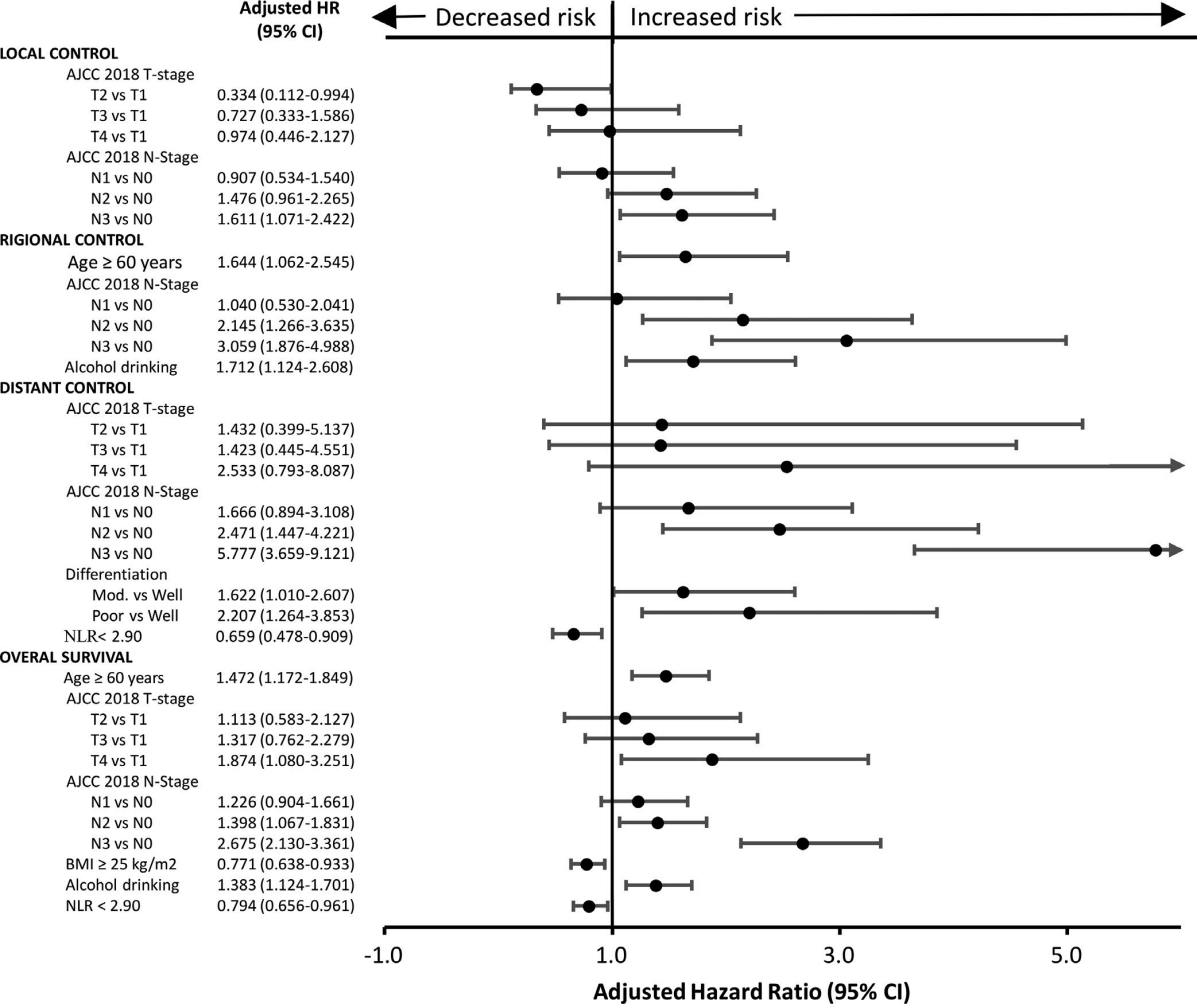
Forest plot based on multivariate analysis adjusted hazard ratios (HRs) from Cox regression for local control, regional control, distant control, and overall survival in the entire cohort (*n* = 890). Abbreviations: AJCC, American Joint Committee on Cancer; BMI, body mass index; CI, confidence interval; HR, hazard ratio; NLR, neutrophil‐to‐lymphocyte ratio

**FIGURE 4:**
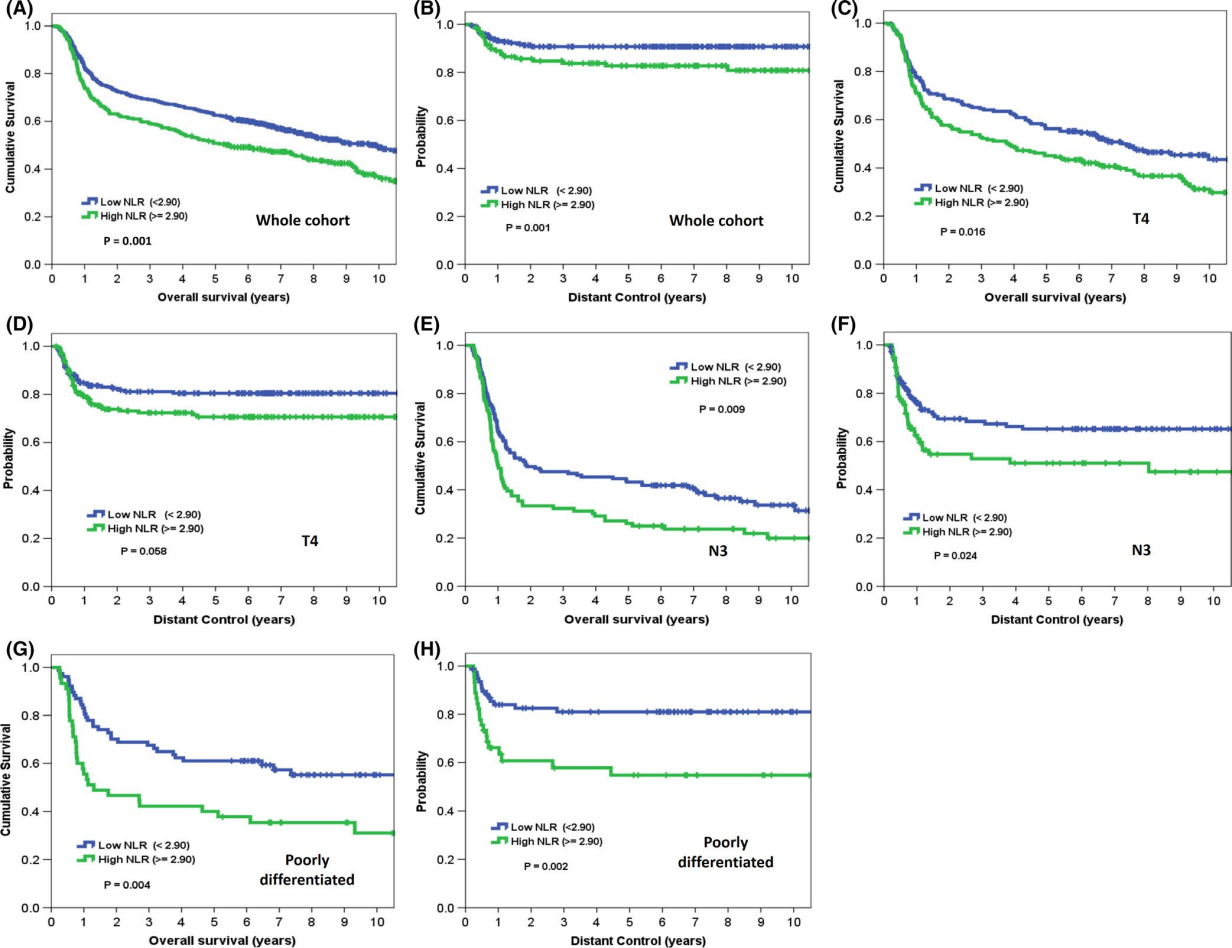
Kaplan‐Meier curves of the study patients stratified according to neutrophil‐to‐lymphocyte ratio (NLR). (A) Overall survival and (B) distant control in the entire study cohort (*n* = 890); (C) overall survival and (D) distant control in patients with T4 disease (*n* = 377); (E) overall survival and (F) distant control in patients with N3 disease (*n* = 237); (G) overall survival and (H) distant control in patients with poorly differentiated disease (*n* = 122)

The following variables were investigated for their associations with OS in univariate analyses: age, sex, tumor subsites, clinicopathologic factors, BMI, risky oral habits, and complete blood count cell ratios (Table 2 and Figure 2D). Variables with univariate associations at a *p* < 0.2 level were entered as covariates in multivariate analyses (Table 3 and Figure 3). Significant univariate adverse risk factors for OS were older age (*p* < 0.01), high T stage (*p* = 0.001), high N stage (*p* < 0.001), high clinical stage (*p* < 0.001), lower BMI (*p* < 0.001), alcohol drinking (*p* = 0.008), treatment with chemotherapy (*p* < 0.001), high NLR (*p* = 0.001) (Figure 4A), and high PLR (*p* = 0.025). In multivariate analyses, a low NLR was independently associated with a more favorable OS (adjusted HR: 0.794, 95% CI: 0.656–0.961, *p* = 0.018, bootstrap *p* = 0.028), regardless of age (*p* = 0.001, bootstrap *p* = 0.001), T4 vs T1 (*p* = 0.025, bootstrap *p* = 0.037), N3 vs N0 (*p* < 0.001, bootstrap *p* = 0.001), N2 vs N0 (*p* = 0.015, bootstrap *p* = 0.011), BMI (*p* = 0.008, bootstrap *p* = 0.014), and alcohol drinking (*p* = 0.002, bootstrap *p* = 0.002). LMR and PLR were not retained in the model as independent risk factors.

**TABLE 2:** Univariate analysis of the four study endpoints

	Local control	*p*	Regional control	*p*	Distant control	*p*	Overall survival	*p*
	HR (95% CI)		HR (95% CI)		HR (95% CI)		HR (95% CI)	
Age ≥60 years	0.926 (0.607–1.414)	0.723	1.430 (0.935–2.189)	0.099	0.875 (0.574–1.332)	0.533	1.339 (1.073–1.670)	0.010
Female sex	1.159 (0.642–2.089)	0.625	1.287 (0.673–2.462)	0.445	0.982 (0.532–1.813)	0.955	1.040 (0.726–1.490)	0.830
Primary tumor site		0.550		0.828		0.839		0.825
Buccal mucosa	1 (Reference)		1 (Reference)		1 (Reference)		1 (Reference)	
Gum	0.883 (0.545–1.431)		0.738 (0.404–1.350)		0.745 (0.454–1.222)		1.027 (0.784–1.344)	
Hard palate	1.520 (0.552–4.187)		0.960 (0.233–3.962)		0.989 (0.310–3.150)		1.350 (0.732–2.488)	
Lip	0.814 (0.255–2.599)		0.764 (0.185–3.156)		0.784 (0.246–2.497)		1.124 (0.611–2.070)	
Mouth floor	0.144 (0.020–1.037)		0.000 (0.000–0.000)		0.711 (0.286–1.768)		0.791 (0.466–1.342)	
Retromolar	0.869 (0.415–1.820)		0.727 (0.288–1.836)		1.117 (0.588–2.122)		0.965 (0.638–1.460)	
Tongue	0.904 (0.624–1.309)		1.144 (0.761–1.719)		0.821 (0.569–1.185)		0.920 (0.739–1.145)	
AJCC 2018 T stage		0.065		0.683		0.012		0.001
T_1_	1 (Reference)		1 (Reference)		1 (Reference)		1 (Reference)	
T_2_	0.335 (0.113–0.997)		0.926 (0.240–3.582)		1.532 (0.427–5.491)		0.924 (0.486–1.755)	
T_3_	0.710 (0.326–1.548)		1.405 (0.440–4.489)		1.554 (0.487–4.955)		1.177 (0.684–2.026)	
T_4_	0.920 (0.424–2.000)		1.430 (0.446–4.585)		2.545 (0.805–9.053)		1.631 (0.950–2.801)	
AJCC 2018 N Stage		0.067		<0.001		<0.001		<0.001
N_0_	1 (Reference)		1 (Reference)		1 (Reference)		1 (Reference)	
N_1_	0.834 (0.494–1.407)		1.163 (0.597–2.262)		1.546 (0.839–2.849)		1.070 (0.795–1.440)	
N_2_	1.353 (0.887–2.603)		2.467 (1.470–4.139)		2.382 (1.414–4.012)		1.269 (0.974–1.653)	
N_3_	1.525 (1.018–2.287)		3.146 (1.938–5.105)		6.190 (3.981–9.623)		2.517 (2.010–3.152)	
AJCC 2018 Stage		0.039		0.086		<0.001		<0.001
I	1 (Reference)		1 (Reference)		0.000 (0.000‐)		1 (Reference)	
II	0.118 (0.012–1.138)		0.000 (0000–9.7518e^199^)		0.196 (0.027–1.403)		0.552 (0.148–2.056)	
III	0.350 (0.107–1.144)		0.750 (0.101–5.574)		0.348 (0.218–0.557)		0.817 (0.300–2.225)	
IV	0.545 (0.173–1.714)		1.391 (0.194–9.980)		1 (Reference)		1.473 (0.550–3.947)	
Differentiation		0.499		0.208		<0.001		0.069
Well	1 (Reference)		1 (Reference)		1 (Reference)		1 (Reference)	
Moderate	1.170 (0.801–1.708)		1.510 (0.940–2.426)		2.025 (1.278–3.209)		1.285 (1.026–1.609)	
Poor	0.894 (0.497–1.608)		1.562 (0.830–2.943)		3.122 (1.820–5.356)		1.338 (0.977–1.832)	
BMI ≥25 kg/m^2^	0.846 (0.613–1.169)	0.310	0.860 (0.595–1.243)	0.421	0.715 (0.518–0.986)	0.041	0.692 (0.574–0.835)	<0.001
Cigarette smoking	1.094 (0.669–1.789)	0.721	0.848 (0.507–1.418)	0.529	1.317 (0.786–2.209)	0.296	0.935 (0.717–1.221)	0.621
Betel quid chewing	1.416 (0.928–2.160)	0.107	0.984 (0.636–1.522)	0.942	1.141 (0.774–1.682)	0.505	1.077 (0.862–1.346)	0.514
Alcohol drinking	1.368 (0.965–1.939)	0.078	1.512 (1.006–2.272)	0.047	1.273 (0.909–1.783)	0.161	1.311 (1.074–1.601)	0.008
Concurrent chemotherapy	1.468 (1.049–2.054)	0.025	1.799 (1.206–2.683)	0.004	2.078 (1.459–2.957)	<0.001	1.489 (1.229–1.804)	<0.001
NLR <2.90	0.913 (0.655–1.272)	0.591	0.923 (0.633–1.346)	0.679	0.586 (0.429–0.801)	0.001	0.720 (0.598–0.867)	0.001
LMR ≥4.21	1.008 (0.728–1.396)	0.962	0.844 (0.578–1.233)	0.380	0.722 (0.528–0.987)	0.041	0.838 (0.696–1.008)	0.061
PLR <110.6	1.310 (0.936–1.832)	0.115	0.879 (0.603–1.282)	0.503	0.662 (0.473–0.928)	0.017	0.804 (0.664–0.972)	0.025

Abbreviations: AJCC, American Joint Committee on Cancer; BMI, body mass index; CI, confidence interval; HR, hazard ratio; LMR, lymphocyte‐to‐monocyte ratio; NLR, neutrophil‐to‐lymphocyte ratio; PLR, platelet‐to‐lymphocyte ratio.

**TABLE 3:** Multivariate analysis of the four study endpoints

	Local control			Regional control			Distant control			Overall survival		
	Adjusted HR (95% CI)	*p*	Bootstrap *p*	Adjusted HR (95.0% CI)	*p*	Bootstrap *p*	Adjusted HR (95.0% CI)	*p*	Bootstrap *p*	Adjusted HR (95% CI)	*p*	Bootstrap *p*
Age ≥60 years				1.644 (1.062–2.545)	0.026	0.055				1.472 (1.172–1.849)	0.001	0.001
AJCC 2018 T stage		0.045						0.004			0.001	
T_1_	1 (Reference)						1 (Reference)			1 (Reference)		
T_2_	0.334 (0.112–0.994)	0.049	0.0280.				1.432 (0.399–5.137)	0.582	0.544	1.113 (0.583–2.127)	0.746	0.755
T_3_	0.727 (0.333–1.586)	0.423	0.387				1.423 (0.445–4.551)	0.552	0.531	1.317 (0.762–2.279)	0.324	0.349
T_4_	0.974 (0.446–2.127)	0.947	0.956				2.533 (0.793–8.087)	0.117	0.079	1.874 (1.080–3.251)	0.025	0.037
AJCC 2018 N stage		0.042			<0.001			<0.001			<0.001	
N_0_	1 (Reference)			1 (Reference)			1 (Reference)			1 (Reference)		
N_1_	0.907 (0.534–1.540)	0.718	0.715	1.040 (0.530–2.041)	0.739	0.743	1.666 (0.894–3.108)	0.108	0.124	1.226 (0.904–1.661)	0.190	0.196
N_2_	1.476 (0.961–2.265)	0.075	0.075	2.145 (1.266–3.635)	0.001	0.002	2.471 (1.447–4.221)	0.001	0.001	1.398 (1.067–1.831)	0.015	0.011
N_3_	1.611 (1.071–2.422)	0.022	0.03	3.059 (1.876–4.988)	<0.001	0.001	5.777 (3.659–9.121)	<0.001	0.001	2.675 (2.130–3.361)	<0.001	0.001
Differentiation								0.021				
Well							1 (Reference)					
Moderate							1.622 (1.010–2.607)	0.046	0.056			
Poorly							2.207 (1.264–3.853)	0.005	0.003			
BMI ≥25 kg/m^2^										0.771 (0.638–0.933)	0.008	0.014
Alcohol drinking				1.712 (1.124–2.608)	0.012	0.021				1.383 (1.124–1.701)	0.002	0.002
NLR <2.90							0.659 (0.478–0.909)	0.011	0.015	0.794 (0.656–0.961)	0.018	0.028
LMR ≥4.21												
PLR <110.6												

Abbreviations: AJCC, American Joint Committee on Cancer; BMI, body mass index; CI, confidence interval; HR, hazard ratio; LMR, lymphocyte‐to‐monocyte ratio; NLR, neutrophil‐to‐lymphocyte ratio; PLR, platelet‐to‐lymphocyte ratio.

### 3.4 Preoperative complete blood count cell ratios and local, regional, and distant control

The following variables were investigated for their associations with LC, RC, and DC in univariate analyses: age, sex, tumor subsites, clinicopathologic factors, BMI, risky oral habits, and complete blood count cell ratios (Table 2 and Figure 2A, B, and C). Variables with univariate associations at a *p* < 0.2 level were entered as covariates in multivariate analyses (Table 3 and Figure 3). A higher clinical stage (*p* = 0.039) and treatment with concurrent chemotherapy (*p* = 0.025) were significant univariate adverse predictors of LC. Multivariate analyses identified T1 vs T2 (*p* = 0.049, bootstrap *p* = 0.028), N3 vs N0 (*p* = 0.022, bootstrap *p* = 0.03) as independent adverse predictors of LC. A higher N stage (*p* < 0.001), alcohol drinking (*p* = 0.047), and treatment with concurrent chemotherapy (*p* = 0.004) were significant univariate adverse predictors of RC. Multivariate analyses identified N3 vs N0 (*p* < 0.001, bootstrap *p* = 0.001), N2 vs N0 (*p* = 0.001, bootstrap *p* = 0.002), and alcohol drinking (*p* = 0.012, bootstrap *p* = 0.021) as independent adverse predictors of RC. None of the three preoperative complete blood count cell ratios were independent predictors of LC or RC.

A higher T stage (*p* = 0.012), N stage (*p* < 0.001), clinical stage (*p* < 0.001), poor differentiation (*p* < 0.001), lower BMI (*p* = 0.041), alcohol drinking (*p* = 0.008), treatment with chemotherapy (*p* < 0.001), high NLR (*p* = 0.001) (Figure 4B), low LMR (*p* = 0.041), and high PLR (*p* = 0.017) were significant univariate adverse predictors of DC. Multivariate analyses identified a low NLR as independently associated with a better DC (adjusted HR: 0.659, 95% CI: 0.478–0.909, *p* = 0.011, bootstrap *p* = 0.015), regardless of N3 vs N0 (*p* < 0.001, bootstrap *p* = 0.001), N2 vs N0 (*p* = 0.001, bootstrap *p* = 0.001), and poor differentiation vs well differentiation (*p* = 0.005, bootstrap *p* = 0.003). Neither LMR nor PLR were independent predictors of DC.

### 3.5 Subgroup analyses in high‐risk patients

Because a high NLR was the blood count cell ratio most consistently associated with adverse outcomes, we performed subgroup analyses of this variable in different subgroups of patients bearing risk factors for distant metastases or death (T4 disease, N3 disease, and poor differentiation). A high NLR was significantly associated with a less favorable OS in all high‐risk subgroups (all *p* < 0.02; Figure 4C, E, and G). Similarly, a high NLR was associated with less favorable DC in patients with N3 disease or poor differentiation (all *p* < 0.03; Figure 4F and H) but not in those with T4 disease (*p* = 0.058; Figure 4D).

## 4 DISCUSSION

In this study, we compared the prognostic value of different preoperative complete blood count cell ratios in patients with OSCC who were treated with radical surgery and PORT. Our results indicate that NLR was superior to both LMR and PLR in the prediction of OS and DC. Notably, NLR retained its statistical significance even in specific subgroups of high‐risk patients, suggesting that it may further refine prognostic stratification with respect to traditional risk factors for poor OS and DC (T4 disease, N3 disease, and poor differentiation).

The exact mechanisms whereby NLR has a higher predictive value than LMR and PLR remain to be established. In general, white blood cell and platelet counts reflect an individual's systemic and/or local inflammatory status. Neutrophils are known to produce cytokines, chemokines, and growth factors that may promote angiogenesis as well as tumor cell proliferation and migration.^13^ Numerous studies have consistently shown that an increased neutrophil count predicts adverse outcomes in patients with different solid cancers.^14^, ^15^, ^16^ In contrast, lymphocytes are responsible for antitumor‐specific immune response—including T‐lymphocyte tumor infiltration^17^ and cytotoxic T‐lymphocyte‐mediated antitumor activity.^18^ Notably, a low lymphocyte count is a poor prognostic factor in patients with malignancies.^19^, ^20^ Platelets produce growth factors that promote cancer growth and its distant spread.^21^, ^22^ A high platelet count predicts unfavorable outcomes in patients with head and neck malignancies, and antiplatelet agents may have a therapeutic antitumor potential.^23^ Finally, monocytes—which can differentiate into tumor‐infiltrating macrophages and dendritic cells—produce proinflammatory molecules involved in carcinogenesis and tumor metastasis.^4^, ^24^ In this regard, a high monocyte count has an adverse prognostic significance in patients with oral cavity cancer.^25^, ^26^, ^27^ Based on these observations, it is not surprising that high NLR and PLR and a low LMR have been related to increased cancer‐related mortality and recurrence rates.^28^, ^29^, ^30^, ^31^

Albeit being the most widely applied tool for predicting prognosis in patients with OSCC, the TNM staging system is a static instrument that solely relies on tumor‐related characteristics. In this scenario, there is an urgent need for reliable prognostic tools grounded on simple preoperative variables. Our results clearly indicate that the preoperative NLR is a simple and effective index that warrants further scrutiny in OSCC. However, the optimal cutoff point for NLR may be population dependent. Cristina et al.^32^ have shown that NLR predicts OS in patients with OSCC – with a cutoff (2.9) in line with our current findings. The question as to whether the same value applies to ethnically different populations remains open.

The incidence of DM in OSCC (approximately 10%) is lower than that observed in other head and neck tumors.^33^, ^34^ Although their presence portends a dismal prognosis, their screening is still not routinely performed. Risk factors for DM in OSCC include histologic grade, number of positive nodes, extracapsular extension, and pT stage.^33^, ^34^, ^35^, ^36^ Our results confirm and expand previous data by showing that NLR is an independent risk factor for DC. Here, we demonstrate that the 1‐ and 5‐year DC rates were 88.7%/84.5% and 82.9%/74.7% in the low and high NLR groups, respectively. Because distant spread occurred more frequently in the first postsurgical year, we believe that strict follow‐up schedules in patients with OSCC should be implemented as early as possible. DM tended to occur early even in high‐risk subgroups (patients with T4 disease, N3 disease, and poor differentiation), further supporting the clinical importance of early screening. Notably, early metastases do not generally shown a disseminated pattern—making them potentially amenable to local salvage attempts (e.g., metastatectomy or stereotactic body radiotherapy).^37^, ^38^ Besides being a simple screening tool for early DM, NLR may potentially serve as a biomarker to stratify the allocation of patients with locally advanced OSCC to treatment intensification strategies (posttreatment metronomic adjuvant chemotherapy).^39^

Our findings need to be interpreted in the context of some limitations. First, our study shares the caveats of retrospective research. However, our sample size was large and allowed a statistically sound comparison of different preoperative complete blood count cell ratios. Second, we did not resort to serial measurements and we do not know whether treatment‐ or time‐induced changes in NLR may modify its prognostic value. Third, all of the study patients underwent radical surgery followed by PORT—indicating that patients with stage III–IV were largely predominant (97%). The question as to whether our findings are generalizable to early‐stage OSCC remains open. Fourth, although our findings were internally validated using the bootstrap method, our findings and optimal NLR cutoff value applying to different cohorts remain answered and need further international validation effort.

In conclusion, a high pretreatment NLR was an independent unfavorable risk factor for both OS and DC in patients with OSCC who underwent surgery and PORT. No other preoperative complete blood count parameters and cell ratios were found to have prognostic significance. Pending independent validation, NLR may serve as a screening tool for DM and to guide patient allocation to treatment intensification strategies.

## CONFLICT OF INTEREST

None.

## Supporting information

Table S1‐S2

## Data Availability

The data that support the findings of this study are available on request from the corresponding author. The data are not publicly available due to privacy or ethical restrictions.
