# Supplementary material for: Comparative prognostic value of different preoperative complete blood count cell ratios in patients with oral cavity cancer treated with surgery and postoperative radiotherapy
Source: Cancer Med. 2021 Feb 23;10(6):1975–88. doi: 10.1002/cam4.3738 (PMC7957172; doi:10.1002/cam4.3738)
Supplement: Supplementary file 1 — Table S1‐S2 [file CAM4-10-1975-s001.docx]

**Appendix**

Supplement Table S1. Maximal AUC value for different time points

| **Time (years)** | **Number of cases** | **Surviving patients** | **Censored patients** | **AUC (LMR)** | **AUC (NLR)** | **AUC (PLR)** |
| --- | --- | --- | --- | --- | --- | --- |
| **1** | 182 | 707 | 1 | 0.5501041 | 0.5415151 | 0.508572 |
| **2** | 275 | 614 | 1 | 0.5416554 | 0.5367675 | 0.5234997 |
| **3** | 307 | 582 | 1 | 0.5467632 | 0.5393251 | 0.5258462 |
| **4** | 337 | 551 | 2 | 0.5466877 | 0.5437766 | 0.5316707 |
| **5** | 371 | 516 | 3 | 0.5399039 | 0.5414571 | 0.5419715 |
| **6** | 389 | 457 | 44 | 0.5515197 | 0.5387805 | 0.5456336 |
| **7** | 409 | 339 | 142 | 0.5605971 | 0.5364324 | 0.5419738 |
| **8** | 427 | 259 | 204 | 0.5811256 | 0.5261538 | 0.5350534 |
| **9** | 438 | 186 | 266 | 0.5717073 | 0.5070807 | 0.5234203 |
| **10** | 450 | 127 | 313 | 0.5972443 | 0.5283171 | 0.5416843 |

Supplement Table S2. Best cutoff value by 1,3,5 year time points

| **Time (years)** | **LMR** | **NLR** | **PLR** |
| --- | --- | --- | --- |
| **1** | 4.18 | 2.95 | 97.75 |
| **3** | 4.21 | 2.85 | 118.92 |
| **5** | 4.21 | 2.90 | 110.62 |
